# Evaluation of *Cynara cardunculus* L. and municipal solid waste compost for aided phytoremediation of multi potentially toxic element–contaminated soils

**DOI:** 10.1007/s11356-020-10687-2

**Published:** 2020-09-10

**Authors:** Matteo Garau, Paola Castaldi, Giacomo Patteri, Pier Paolo Roggero, Giovanni Garau

**Affiliations:** 1 Dipartimento di Agraria, University of Sassari, Viale Italia 39, 07100 Sassari, Italy; 2 Nucleo di Ricerca sulla Desertificazione, University of Sassari, Viale Italia 39, 07100 Sassari, Italy

**Keywords:** Potentially toxic elements, Cardoon, Organic amendments, Bioaccumulation, Translocation factor, Community-level physiological profile, Soil enzyme activities

## Abstract

The suitability for aided phytoremediation of *Cynara cardunculus* L. var. *altilis* and municipal solid waste compost (MSWC) applied at 2% and 4 % rates was evaluated in a multi potentially toxic element (PTE)-contaminated mining soil (Pb ~ 15,383 mg kg^−1^, Zn ~ 4076 mg kg^−1^, As ~ 49 mg kg^−1^, Cd ~ 67 mg kg^−1^, Cu ~ 181 mg kg^−1^, and Sb ~ 109 mg kg^−1^). The growth of *C. cardunculus* significantly increased with compost amendment and followed the order: MSWC-4% > MSWC-2% > Control. PTE concentrations in the roots of plants grown on amended soils decreased compared with control plants (i.e., less than ~ 82, 94, and 88% for Pb, Zn, and Cd respectively). PTE translocation from roots to shoots depended on both PTE and amendment rate but values were generally low (i.e., < 1). However, PTE mineralomasses were always higher for plants grown on MSWC-amended soils because of their higher biomass production, which favored an overall PTE bioaccumulation in roots and shoots. After plant growth, labile As and Sb increased in amended soils, while labile Pb, Zn, Cu, and Cd significantly decreased. Likewise, dehydrogenase and urease activities increased significantly in planted soils amended with MSWC. Also, the potential metabolic activity and the catabolic versatility of soil microbial communities significantly increased in planted soils amended with MSWC. Overall, our results indicate that *C. cardunculus* and MSWC can be effective resources for the aided phytoremediation of multi PTE-contaminated soils.

**Electronic supplementary material:**

The online version of this article (10.1007/s11356-020-10687-2) contains supplementary material, which is available to authorized users.

## Introduction

Mining activities usually release in the environment high amounts of potentially toxic elements (PTE) like Cd, Cu, Pb, Zn, As, and Sb, especially because of the absence of effective recovery and securing interventions. This in turn can produce severe and widespread pollution of soil and ground water in large areas or entire regions (Castaldi et al. 2018; Cidu et al. 2012; Garau et al. 2017).

It is widely accepted that environmental pollution by PTE represents a serious problem which involves both environmental and human health implications. PTEs have been identified as responsible for DNA damage and carcinogenicity, as well as neurological and cardiovascular problems (Wuana and Okieimen 2011). Elevated PTE concentrations in soil can negatively affect plant growth, food and feed quality, and the abundance, diversity, and activity of soil (micro)biota, which are in turn responsible for element cycling and soil organic matter turnover among the others (Castaldi et al. 2018; Garau et al. 2019a, 2019b; Renella et al. 2005).

Some chemical and physical treatments to contaminated soils can irreversibly modify the properties of soil, destroying its (micro)biota and making it inhospitable to plants (Padmavathiamma and Li 2007). Several studies have been addressed on the development of novel and low-impact techniques for the remediation of PTE-contaminated soils (e.g., Garau et al. 2014; Mench et al. 2006). Particular attention has been paid on different in situ remediation approaches, such as PTE immobilization and phytoremediation, which are considered low-cost and ecologically sustainable interventions (Wuana and Okieimen 2011). Phytoremediation, which exploits the ability of plants to stabilize, absorb, transfer, and/or degrade the contaminants within soil or water, can be considered a promising green approach for the recovery of soils polluted with PTE (Ali et al. 2013; Gomes et al. 2016). Moreover, the use of plant species with phytoremediation potentials, which can be also used to produce bioenergy, e.g., heat and/or electricity, could be an option for the reclamation of PTE-polluted soils that in any case cannot be used for food or feed production.

To date, the exploitation of not renewable energy sources has substantially contributed to environmental pollution (Destek and Aslan 2017), while plant growth for bioenergy production has been identified as a strategy to reduce the consumption of fossil fuels and preserve the environment. However, this practice is in competition for food and/or feed production in fertile soils (Aslani et al. 2018; Destek and Aslan 2017). The use of PTE-contaminated soils to cultivate bioenergy crops with phytoremediation capacity could contribute to green energy production and soil remediation, without affecting the agricultural areas devoted to food and/or feed production (Domínguez et al. 2017). Ideally, plants selected for this purpose should be characterized by low input requirements (e.g., fertilization), suitable biomass production, and ability to further stabilize labile contaminants in soil, in order to ensure both an economic and environmental sustainability (Pandey et al. 2016).

Plants developed different mechanisms in order to survive in soils with high concentrations of PTE, e.g., stabilizing the contaminant at the soil-root interface and/or immobilizing them in belowground organs (e.g., roots) or in soil (Barbosa and Fernando 2018). This is the basis of phytostabilization, a type of phytoremediation in which plants are used to stabilize wastes, prevent wind and water soil erosion, limit the leaching of pollutants into groundwater, and immobilize pollutants by means of physical and/or chemical processes (Sinhal et al. 2015). Importantly, the effectiveness of phytostabilization can be enhanced by the addition to soil of different sorbent materials such as compost, biochar, water treatment residues or others, able to decrease the bioavailability of PTE and improve the soil physico-chemical and biological properties (Bacchetta et al. 2015; Garau et al. 2014; Manzano et al. 2016). Such approach, which is referred to as aided or assisted phytostabilization, has attracted considerable interest in the last decade and studies evaluating its efficacy are constantly growing (e.g., Alvarenga et al. 2008; Castaldi et al. 2018).

Different plant species can be used in aided phytoremediation of PTE-contaminated soils, some of which have been also selected as bioenergy crops (Pandey et al. 2016). Several studies have focused on cardoon (*Cynara cardunculus* L.) for its rusticity, productive attitudes, and potential use in phytoremediation programs (Arena et al. 2017; Domínguez et al. 2017; Pandey et al. 2016; Papazoglou 2011). Previous studies demonstrated that cardoon plants were tolerant to Cd and Pb in contaminated soils by increasing the Rubisco synthesis to compensate for damage to chloroplasts and to preserve the photosynthesis efficiency (Arena et al. 2017; Sorrentino et al. 2018). Other studies showed that cardoon behaved as PTE excluder in As contaminated soils and as PTE accumulators in Cd or Pb polluted ones (Hernández-Allica et al. 2007; Papazoglou 2011; Sánchez-Pardo et al. 2015). Finally, Domínguez et al. (2017) showed that cardoon biomass was not significantly influenced by the presence of PTE in soil, while its yield rapidly decreased in acidic soils.

In a previous study, we evaluated the influence of municipal solid waste compost (MSWC) on the mobility, toxicity, and bioaccessibility of PTE in a contaminated soil (Garau et al. 2019a). In this study, we tested the hypothesis that MSWC can be used in aided phytoremediation programs using cardoon. Research to understand the effectiveness of such plant-amendment combination in PTE-contaminated soils is particularly needed, especially if we consider that (to the best of our knowledge) only one recent study partially addressed this topic using *C. cardunculus* and a biosolid compost in an environment characterized by a relatively low PTE contamination (Madejón et al. 2019). Therefore, this study focuses on the growth of *C. cardunculus* in a highly PTE-polluted soil treated (or untreated) with MSWC and PTE uptake, bioaccumulation and translocation in the plant. The hypothesis is that the combination of soil amendment and cardoon cultivation can provide a viable option for aided phytoremediation programs. The PTE mobility in soil was also quantified following the cultivation of cardoon and soil biological fertility parameters (e.g., community-level physiological profile, dehydrogenase, urease, and β-glucosidase activities) were assessed.

## Materials and methods

### Experimental setup and plant analysis

Soil samples were collected near the ex-mining area of Montevecchio (Guspini) in the Southwestern Sardinia (39.559813N, 8.5915124E, Montevecchio-Levante). Galena (PbS) and blenda (ZnS) were the main ores extracted (Romano et al. 2017). Soil samples were randomly collected from the upper 25 cm soil over approx. 1 ha, bulked together in the laboratory and used to set up different mesocosms (approx. 15 kg each) which received distinct treatments. The main features of the (bulked) soil used for mesocosms set up were previously reported (Table S1; Garau et al. 2019a). Briefly, the soil was a sandy clay loam (USDA classification) with a bulk density of 1.32 g cm^−3^ (ISO 11272: 2017), it had a sub-acidic pH (i.e., 5.93), a substantial content of organic matter (OM, ~ 3.2%), and a high cation exchange capacity (CEC, 36.86 cmol_(+)_ kg^−1^) (Garau et al. 2019a). The total concentration of Pb (15,383 mg kg^−1^), Zn (4076 mg kg^−1^), Cu (181.4 mg kg^−1^), Sb (109.5 mg kg^−1^), Cd (67.45 mg kg^−1^), and As (48.75 mg kg^−1^) exceeded the threshold levels established by the Italian legislation (Dlgs. 152/2006). In addition, PTE concentrations in soils was exceeding the threshold values defined by the Finnish legislation (Government Decree on the Assessment of Soil Contamination and Remediation, Needs 214/2007), which represent a satisfying approximation of the mean values of different European countries (Tóth et al. 2016).

The following treatments were applied to soil mesocosms: polluted untreated soil (Control); 2% (w/w) MSWC (MSWC-2%); 4% (w/w) MSWC (MSWC-4%). The MSWC was derived from municipal and green waste composting and was provided by the Facility Plant Secit S.p.A. Consorzio Zir (Chilivani-Ozieri, Italy). The main chemical characteristics of the MSWC are reported in Table S1 and carefully described by Diquattro et al. (2018). Briefly, the compost had a subalkaline pH (i.e., 7.93), a total organic carbon content (27.3% d.m.) in line with values reported in literature for composts obtained from municipal solid wastes, a high cation exchange capacity (CEC, 92.3 cmol_(+)_ kg^−1^), dissolved organic carbon (DOC, 0.82 mg kg^−1^ d.m.), and humic acids content (14.24 % d.m.).

All mesocosms were incubated for 4 months at 20 °C and 30% of their water holding capacity. Afterwards, a total of 18 pots (19 cm diameter, 17 cm height) each containing 1.5 kg of soil deriving from the different mesocosms were set up, i.e., 6 replicated pots x 3 treatments x 1 plant species. Six cardoon seeds (*C. cardunculus* L.) cv. *altilis* (provided by Lovelegis, Salerno, Italy) were then planted in each pot within a week after the soil/amendment incubation period. Since all seeds successfully germinated, after 2 weeks growth, cardoon plants were thinned to 4 per pot (24 plants for treatment) and grown until the end of the experiment. Planted pots were arranged in a completely randomized design and plants were grown over 5 months in a greenhouse under controlled conditions (20–25 °C temperature, 60–70% relative humidity, and ~ 16,400 kJ m^−2^ mean global radiation). The experiment was stopped at the 5^th^ month when control plants started to show substantial signs of suffering. At the end of the growth phase, a visual exam of the aerial part development was carried out.

At harvest, shoots were separated from roots, carefully washed with deionized water and dried at 55 °C for 72 h. The average dry weight, PTE concentration, bioaccumulation and translocation factors and mineralomasses were determined in such shoots and roots.

PTE concentration was determined after mineralization of the plant material with 2 mL suprapure H_2_O_2_ and 9 mL of HNO_3_ and ultrapure H_2_O (ratio 1:1), using a Microwave Milestone MLS 1200. The total concentration of PTE (i.e., Pb, Cd, Zn, Cu, As, and Sb) was determined using a Perkin Elmer Analyst 200 flame atomic absorption spectrometer (AAS, for Zn and Cu quantification) or a Perkin Elmer AAnalyst 400 atomic adsorption spectrometer equipped with a graphite furnace (GAAS, for Pb, Cd, As, and Sb quantification). Peach leaves were used as standard reference material (NIST-SRM 1515). Three replicate samples per treatment were analyzed and mean values ± standard deviations are reported.

The PTE bioaccumulation (BAF) and translocation (TF) factors and mineralomasses (MM) were calculated as follows (Table 1; Sánchez-Pardo et al. 2015):

- BAF_R_ was calculated as the ratio between the PTE concentration in roots to that present in soil;
- BAF_S_ was calculated as the ratio between the PTE concentration in shoots to that present in soil;
- TF was calculated as the ratio between the PTE concentration in shoots to that present in roots.

**Table 1:** Bioaccumulation (BAF_R_ and BAF_S_) and translocation (TF) factors and mineralomasses (MM_R_ and MMs) of PTE in *C. cardunculus*. Values represent mean ± SD (*n* = 3). For each PTE, BAF, TF, and MM values followed by different letters indicate statistically significant differences according to the Tukey multiple comparison test (*P* < 0.05)

		BAF_R_	BAF_S_	TF	MM_R_ (μg)	MM_S_ (μg)
As	Control	0.26^c^	0.05^c^	0.19^a^	1.32^a^	0.41^a^
	MSW-C2%	0.02^a^	0.02^b^	0.86^b^	2.01^b^	0.76^b^
	MSW-C4%	0.03^b^	0.01^a^	0.28^a^	3.63^c^	0.72^b^
Cd	Control	0.57^b^	1.69^b^	2.93^a^	0.41^a^	3.92^a^
	MSW-C2%	0.07^a^	0.30^a^	4.49^b^	7.99^b^	15.84^b^
	MSW-C4%	0.07^a^	0.21^a^	2.82^a^	11.67^c^	23.46^c^
Cu	Control	0.34^c^	0.11^b^	0.32^ab^	6.46	3.34^a^
	MSW-C2%	0.09^a^	0.05^a^	0.57^b^	34.81^b^	8.73^b^
	MSW-C4%	0.11^b^	0.04^a^	0.34^a^	50.47^c^	12.28^b^
Pb	Control	0.13^b^	0.03^b^	0.20^a^	210^a^	60^a^
	MSW-C2%	0.02^a^	0.01^a^	0.33^b^	670^b^	90^b^
	MSW-C4%	0.03^a^	0.01^a^	0.24^ab^	890^c^	150^c^
Sb	Control	0.11^b^	0.01^b^	0.09^a^	1.12^a^	0.16^a^
	MSW-C2%	0.02^a^	0.01^a^	0.44^b^	3.01^b^	0.58^b^
	MSW-C4%	0.02^a^	0.01^a^	0.39^b^	4.13^c^	1.16^c^
Zn	Control	2.44^b^	1.09^c^	0.45^a^	1020^a^	730^a^
	MSW-C2%	0.12^a^	0.26^b^	2.06^c^	1140^b^	920^b^
	MSW-C4%	0.12^a^	0.15^a^	1.29^b^	1300^c^	1190^c^

Mineralomasses, which quantify the actual amounts of PTE absorbed by the plants and stored in roots (MM_R_) or shoots (MM_S_) (Lebrun et al. 2018), were determined to estimate the PTE removal efficiency of *C. cardunculus*. MM were calculated following the procedure of Lebrun et al. (2018) as:

- MM_R_ = biomass of the roots × PTE concentration in roots;
- MM_S_ = biomass of the shoots × PTE concentration in shoots.

### Soil properties and PTE mobility after plant growth

At harvest, root-adhering soil collected from all the pots deriving from the same mesocosm was bulked together, sieved to < 2 mm, and chemically characterized. In particular, soil pH (ISO 10390 2005), electric conductivity (EC; ISO 11265 1994), total organic C and total N (CHN analyzer Leco CHN 628; Abou Jaoude et al., 2020), and the dissolved organic carbon (DOC) were determined (Table 2). The DOC was quantified by UV absorbance (254 nm) of filtered (45 μm) soil suspension as previously described (Brandstetter et al. 1996).

**Table 2:** Chemical properties (mean ± st. dev.) of unamended and MSWC-amended soils after plants growth. Values represent mean ± SD (*n* = 3). For each row, mean values followed by different letters indicate statistically significant differences according to the Tukey multiple comparison test (*P* < 0.05)

	Control	MSWC-2%	MSWC-4%
pH_H2O_	6.18 ± 0.07^a^	6.88 ± 0.00^b^	7.08 ± 0.01^c^
EC (μS cm^−1^)	798 ± 2.31^b^	827.5 ± 6.08^b^	831 ± 2.31^b^
Total organic matter (%)	2.79 ± 0.05^a^	3.99 ± 0.14^b^	4.66 ± 0.16^c^
Total N (%)	0.10 ± 0.00^a^	0.18 ± 0.005^b^	0.21 ± 0.008^c^
Total C (%)	1.62 ± 0.03^a^	2.31 ± 0.08^b^	2.70 ± 0.10^c^
DOC (mg g^−^1)	0.07 ± 0.00^a^	0.32 ± 0.00^b^	0.35 ± 0.01^c^
Total PTE			
Total As	48.65 ± 1.91^a^	46.87 ± 0.71^a^	47.94 ± 0.93^a^
Total Cd	69.81 ± 3.46^a^	64.93 ± 4.07^a^	63.88 ± 0.53^a^
Total Cu	185.77 ± 11.42^a^	199.57 ± 7.65^a^	190.43 ± 10.63^a^
Total Pb	16,108 ± 55.19^a^	16,185 ± 337.52^a^	15,810 ± 315.36^a^
Total Sb	102.48 ± 5.00^a^	104.8 ± 1.97^a^	101.18 ± 1.42^a^
Total Zn	4034 ± 11.463^a^	4035 ± 10.80^a^	4080 ± 15.44^a^

The mobility of Cd, Cu, Pb, and Zn in the root-adhering soil was determined by sequential extraction following the procedure of Basta and Gradwohl (2000). Soil samples (1 g) were firstly treated with 25 mL of a 0.5 M Ca(NO_3_)_2_ solution and shaken for 16 h at 20 °C, to extract the Me(II)-exchangeable pool (Fraction 1); then, the same soil samples were shaken for 5 h at 20 °C with 25 mL of a 1 M NaOAc solution at pH 5.0, to extract the Me(II) forming weak surface complexes (Fraction 2) and finally with 25 mL of a 0.1 M Na_2_EDTA solution for 6 h at 20 °C to extract the Me(II) surface complexed and/or precipitated (Fraction 3).

The mobility of As and Sb in the root-adhering soil was determined following the sequential extraction procedure of Wenzel et al. (2001). In particular, soil samples (1 g) were treated with 25 mL of a 0.05 M (NH_4_)_2_SO_4_ solution and shaken for 4 h at 20 °C to extract the non-specifically sorbed As and Sb (Fraction 1); then, the same samples were treated with 25 mL of a 0.05 M NH_4_H_2_PO_4_ solution and shaken for 16 h at 20 °C h to extract the specifically sorbed PTE (Fraction 2). The same soil samples were subsequently treated with 25 mL of 0.2 M NH_4_^+^-oxalate buffer, shaken for 4 h in the dark at 20 °C to extract the PTE associated with amorphous and poorly crystalline hydrous oxides of Fe and Al (Fraction 3). Finally, soil samples were treated with 25 mL of 0.2 M NH_4_^+^-oxalate buffer + 0.1 M ascorbic acid and shaken for 0.5 h in a water basin at 96 °C to extract the PTE associated with well-crystallized hydrous oxides of Fe and Al (Fraction 4).

In both procedures, following each extraction step, soil samples were centrifuged at 3500 rpm for 15 min and filtered (Whatman 41 filters) to separate the liquid and solid phases. After the last step of the sequential extraction, the residual fraction of PTE was determined by treating soil samples with 3 mL H_2_O_2_ suprapure, 12 mL of HNO_3_ and HCl (3:1 ratio), and digestion in a Microwave Milestone MLS 1200. The PTE concentration in the liquid phase of each extraction step was determined as previously reported.

Each experiment was conducted in triplicate and mean values ± standard deviations are reported.

### Soil enzyme activities and community-level physiological profile after plant growth

Fresh root-adhering soil samples collected from all the pots deriving from the same mesocosm were bulked together, sieved to < 2 mm, and used to investigate selected biochemical and microbiological features. The dehydrogenase activity (DHG) was determined in soil samples added with triphenyltetrazolium chloride and incubated at 37 °C for 24 h (Alef and Nannipieri 1995). The urease activity (URE) was determined as ammonia released in soil samples treated with urea and incubated for 2 h at 37 °C, while the β-glucosidase activity (GLU) was quantified as p-nitrophenol released in soil samples added with p-nitrophenyl glucoside and incubated for 1 h at 37 °C (Alef and Nannipieri 1995).

The community-level physiological profile (CLPP) of soil microbial communities was determined using Biolog EcoPlates (Biolog Inc., Hayward, CA). Microbial communities were extracted from each soil sample and inoculated into the wells of a Biolog EcoPlate following the procedure described by Garau et al. (2011). The Biolog EcoPlate is a 96-wells microtiter plate containing in each well a sole C source; 31 different carbon sources, replicated three times, are present within the plate together with three control wells containing no carbon. A redox dye (tetrazolium violet) is incorporated in each well to reveal oxidative catabolism of the C source (Garau et al. 2011). This latter was quantified for each well every 24 h by recording the optical density at 590 nm (OD_590_) using a Biolog MicroStation microplate reader (Biolog, Hayward, CA). Raw Biolog data (i.e., OD_590_ values) were processed as previously described (Garau et al. 2011), to determine the average well color development (AWCD), which represents the potential metabolic activity of the microbial community, Shannon Index, which represents an indicator of the biological diversity of the community, and Richness, or the number of carbon sources catabolized by the community. These C source utilization data were also analyzed by Principal Component Analysis (PCA) using a correlation matrix to investigate the treatment effects on the structure of soil microbial community (Garland 1997). All Biolog-derived indexes and PCA analysis refers to the 120 h time-point as this incubation time provided the best discrimination among samples. Soil enzyme activities and CLPP were determined in triplicate soil samples and mean values ± standard deviations are reported.

### Data analysis

Plant biomass, PTE bioaccumulation, chemical and microbiological data relative to root-adhering soil were reported as mean values ± standard deviations (SD). Quantification of PTE in plant tissues, and chemical and microbiological analyses of root-adhering soil were performed on triplicate plant or soil samples. One-way Analysis of Variance (One-way ANOVA) was carried out to compare mean values from different treatments. Where significant *P*-values (< 0.05) were obtained, differences between individual means were compared with the post-hoc Tukey multiple comparison test (*P* < 0.05). Statistical analyses were carried out using the NCSS 2007 Data Analysis software (v. 07.1.21; Kaysville, Utah).

## Results and discussion

### Influence of MSWC on cardoon growth in the PTE-contaminated soil

**Fig. 1:**
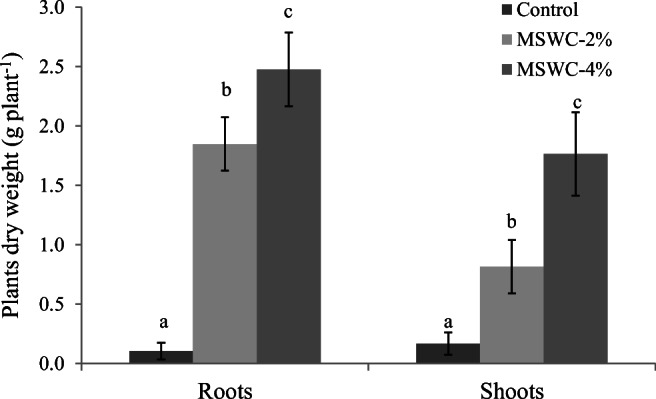
Root and shoot dry weight of *C. cardunculus* grown on amended and unamended soils. Values represent mean ± SD (*n* = 3). For each plant part, mean values followed by different letters denote statistically significant differences according to the Tukey multiple comparison test (*P* < 0.05)

Compost addition to the polluted soil positively and significantly affected the biomass production of *C. cardunculus*. The amendment had substantial influence on root and shoot biomass, which was the highest for plants grown on MSWC-4% (Fig. 1). Root biomass increased by more than 17- and 23-fold in soils amended with 2 and 4% MSWC respectively, compared with unamended (control) soil, while shoot biomass increased by approx. 5- and 10-fold (Fig. 1). Substantial differences were observed on leaves shape and margins at harvest, which were probably due to a delay of the phenological stage induced by PTE (Kozlov et al. 2007). In particular, control plants showed the first rosulate leaves, which were entire with an elliptic shape and crenate margins. The leaf shape of plants grown on 2% MSWC was similar to that recorded for control, but the blade area was more expanded, and the petiole more elongates than control plants. By contrast, the leaves of MSWC-4% plants showed a shape change from elliptic to runcinate with lobed margins. Overall, both compost rates (MSWC-4% in particular) promoted a better phenological development of *C. cardunculus* compared with control soil.

The marked differences in the biomass production were attributed to the PTE-fixing ability of MSWC as previously shown by Garau et al. (2019a), as well as to a better fertility status of the amended soils. Total organic C and N, CEC, available K, Ca, Mg and P and DOC followed the order MSWC-4% > MSWC-2% > control soil (Garau et al. 2019a). A combination of these factors could be responsible for the enhanced plant yield in the amended soils. These data clearly highlight the key role of MSWC in the improvement of cardoon growth in soils heavily contaminated by different PTE. Something similar was recently shown by Madejón et al. (2019), which recorded a significantly higher yield of cardoon plants in a PTE-contaminated area after the addition of 30 tonnes per ha of biosolid compost. The shoot dry weight recorded by Madejón et al. (2019) in control and compost-amended soils was higher than that recorded in our soil (i.e., 10- and 2.7-fold respectively). This was interpreted as the consequence of the much lower contamination status of the soil treated by Madejón et al. (2019) where Pb, Zn, Cu and Cd concentrations were 70-, 25-, 2.5-, and 250-fold lower respectively than in our soil. To our knowledge, no other studies are available on cardoon growth in contaminated amended soils. However, our study was performed in the first five months of cardoon establishment, while the highest cardoon yield generally occurs in the second and third year of growth (Grammelis et al. 2008; Ronga et al. 2019). However, the results suggest that cardoon and MSWC can be a promising combination for the production of plant biomass with different potential uses (e.g., bioenergy production) in PTE-contaminated soils. To test if this combination can be also useful in aided phytoremediation programs, we analyzed the PTE uptake by the plant as well as the PTE bioaccumulation, translocation, and mineralomasses.

### Influence of MSWC on PTE uptake by cardoon plants

**Fig. 2:**
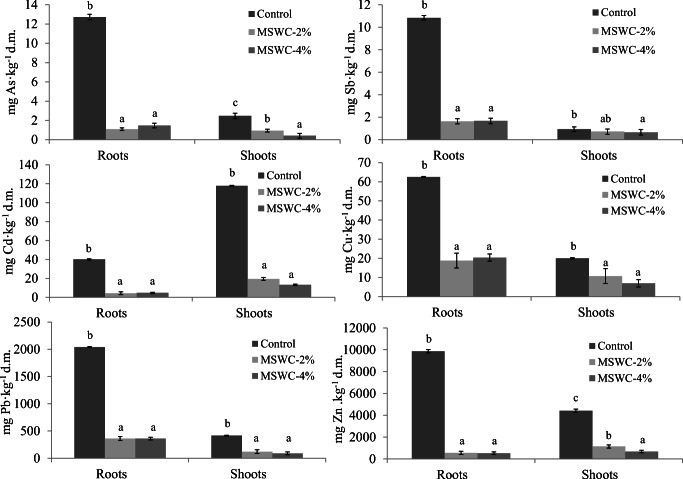
PTE in roots and shoots of *C. cardunculus* grown on the amended and unamended soils. Values represent mean ± SD (*n* = 3). For each plant part, mean values followed by different letters denote statistically significant differences according to the Tukey multiple comparison test (*P* < 0.05)

The MSWC addition reduced significantly the concentration of PTE in plant tissues regardless of the amount added (Fig. 2). The highest concentrations of PTE were generally found in roots, except for Cd whose concentrations were higher in shoots, e.g., up to 5-fold in 2% amended soils (Fig. 2). The As and Sb concentration in roots decreased by 11.7- and 8.7-fold (As), and 6.7- and 6.5-fold (Sb), in 2 and 4% amended soils respectively, compared with control (unamended) roots. Also, the As concentration in shoots was reduced by 2.63- and 6.07-fold in plants grown on 2 and 4% MSWC respectively (compared with control shoots), while the decrease of Sb was less marked but still significant, especially in the case of MSWC-4% (Fig. 2). These data are consistent with the results reported by other studies, which showed that *C. cardunculus* was able to efficiently retain As in roots (Llugany et al. 2012; Sánchez-Pardo et al. 2015). Our results indicate that cardoon behaves similarly with Sb. These data are consistent with the capacity of MSWC to reduce labile As and Sb in soil, while increasing their residual, i.e., hardly bioavailable, pools (Garau et al. 2019a). This also suggests the negligible relevance (if any) of root-induced As and Sb remobilization phenomena occurring in the rhizosphere environment.

Cd, Cu, Pb, and Zn uptake was generally reduced by MSWC addition to soil (Fig. 2). In particular, a noteworthy result was the reduction of Cd concentration in shoots of plants grown on amended soils, ~ 7.5-fold in the amended soils (Fig. 2). This is remarkable, since differently from all the other PTE that we monitored, Cd was highly translocated and bioaccumulated in shoots of control plants, in agreement with the results reported by other authors (Llugany et al. 2012; Papazoglou 2011; Sánchez-Pardo et al. 2015). The reduction of Zn concentration in roots after MSWC addition was also observed (~ 18.4-fold in the amended soils). Likewise As and Sb, the reduced metal uptake by cardoon in amended soils was explained with a substantial fixation of labile metal cations by compost as previously found (Manzano et al. 2016; Silvetti et al. 2017; Garau et al. 2019a).

### Influence of MSWC on PTE bioaccumulation and translocation factors and mineralomasses of cardoon plants

In order to evaluate the phytoremediation (i.e., phytostabilization or phytoextraction) capabilities of *C. cardunculus* in the presence of MSWC, PTE bioaccumulation (i.e., BAF_R_ and BAF_S_) and translocation factors (i.e., TF) were calculated along with mineralomasses (i.e., MM_R_ and MM_S_) for plants growing on amended and unamended soils.

In general, PTE BAF was quite low for plants grown in the unamended soil, i.e., 0.01–0.57 (Table 1). The only exceptions were recorded for Cd (BAF_S_ 1.69) and Zn (BAF_R_ 2.44; BAF_S_ 1.09), that indicated higher concentrations of PTE in plant tissues than those recorded in soil (Table 1). The addition of MSWC significantly reduced PTE bioaccumulation by cardoon plants, and BAF factors relative to all PTE were in the order: control soil > MSWC-2% ≥ MSWC-4%. In particular, BAFs in amended soils were reduced by a minimum of approx. 3-fold (i.e., for Cu) up to a maximum 8-fold (i.e., for Cd) (Table 1). Such PTE bioaccumulation decrease in the amended soils is the logical consequence of the reduced PTE uptake recorded in the presence of MSWC (Fig. 2). Moreover, these data support a stable immobilization of the contaminants by the compost and indicate a negligible influence of root activity on PTE remobilization. This latter aspect, however, needs to be proved.

The selection of plant species for phytoremediation purposes is commonly based on the evaluation of their ability to accumulate PTE and/or their attitude to translocate them from roots to shoots. In order to address this latter point, the TF values relative to all PTE were calculated for cardoon plants grown in amended and unamended soils. The knowledge of such indexes can provide useful information on the potential phytostabilization or phytoextraction capabilities of cardoon. TF values < 1 indicate higher PTE concentration in roots (rather than shoots) which is typical of phytostabilizing species, while TF > 1 indicate higher PTE concentration in shoots (rather than roots) which is typical of phytoextracting species (Barbosa and Fernando 2018).

Cardoon plants grown in the unamended soil showed TF < 1 (i.e., 0.09–0.45) for all the PTE considered except for Cd (TF = 2.93), and TF values followed the order: Cd > Zn > Cu > Pb > As > Sb (Table 1). This indicated that all PTE but Cd, mainly accumulated in roots and were poorly translocated in shoots. This is in agreement with the results reported by other authors which showed low TFs for As, Ni, Pb, and Zn in cardoon plants (Arena et al. 2017; Hernández-Allica et al. 2007; Papazoglou 2011; Sánchez-Pardo et al. 2015; Sorrentino et al. 2018). As a general trend, compost addition increased the TF of all PTE and such increases appeared dependent on the amendment rate. In particular, MSWC-2% consistently increased the TF of all PTE, while MSWC-4% predominantly had little to moderate effect (e.g., As, Cd, Cu, Pb), even if in the case of Sb and Zn, the respective TF were still substantially higher than those of control plants (Table 1). Moreover, TF recorded in all amended soils were still < 1 (i.e., 0.24–0.86) except for Cd, but in this case the TF of control plants was already > 1, and Zn (Table 1). These results suggest that the addition of compost (especially at 4% rate) had limited effects on PTE partitioning between shoots and roots and these latter remained (in the majority of cases) the main sink of PTE for cardoon plants.

The biostimulation effect of the MSWC on cardoon, due to a strong impact of amendment on the soil fertility (Garau et al. 2019a), could have determined the development of adaptive characteristics, favoring the anatomical and physiological response of the plant, such as a higher shoots growth and plants metabolic activity, which resulted in a higher translocation of PTE, in particular as regards Cd and Zn, by shoots tissues (Asemoloye et al. 2017).

The PTE mineralomasses (MM_R_ and MM_S_) were always significantly higher for cardoon plants grown on soils amended with MSWC (Table 1). This indicates that, despite the lower BAF recorded for plants growing in treated soils, these plants showed a higher PTE removal efficiency. This is due to the significantly higher yield of cardoon plants in amended soils, which in turn was attributed to PTE immobilization by the MSWC and/or to an improved fertility of treated soils (Garau et al. 2019a). The MM values for all PTE generally followed the order: Control < MSWC-2% < MSWC-4% (Table 2). It should be noted that MM_R_ and MM_S_ values for plants grown in treated soils were between 1.1–7.8 and 1.5–3.7-fold higher respectively compared with those of control plants. This suggests a good adaptation of cardoon plants to PTE-contaminated soils when polluted soil is amended with compost. Moreover, MM_R_ values in amended soils were significantly higher than MM_S_ ones indicating that all PTE but Cd were preferentially stored within the root system. This could be considered useful from an environmental point of view since most of PTE are immobilized below ground, which limits the risk of contaminant spreading and their entry the food chain.

Taken together, the data indicate that *C. cardunculus* could be used, in combination with MSWC, for the aided phytoremediation (i.e., mainly phytostabilization) of multi PTE-contaminated soils.

### Influence of MSWC and cardoon growth on main soil properties and PTE mobility in the rhizosphere

After the cultivation of cardoon, an increase of soil pH was observed in amended soils (0.7–0.9; Table 2) as well as a higher total organic carbon, nitrogen, and DOC content which followed the order: MSWC-4% > MSWC2% > control soil (Table 2). The PTE mobility after plant growth was also addressed in root-adhering soil to highlight possible PTE remobilization effects due to roots activity. Plants can modify the rizhospheric soil by the uptake of macro- and micro-nutrients and the release of organic or inorganic compounds which can alter the solubility and bioavailability of PTE (Ali et al. 2013; Gomes et al. 2016). In particular, the release of low molecular weight organic compounds such as oxalic, malic and citric acids can increase the solubility of metals (Castaldi et al. 2013; Bothe and Słomka 2017), while the release of polygalacturonic acid can be an effective strategy for the immobilization of metal cations and the reduction of their bioavailability (Castaldi et al. 2010; Kelly-Vargas et al. 2012; Silvetti et al. 2017). Moreover, also rhizospheric microorganisms can influence PTE mobility in soil, e.g., through the release of siderophores (Gomes et al. 2016).

**Fig. 3:**
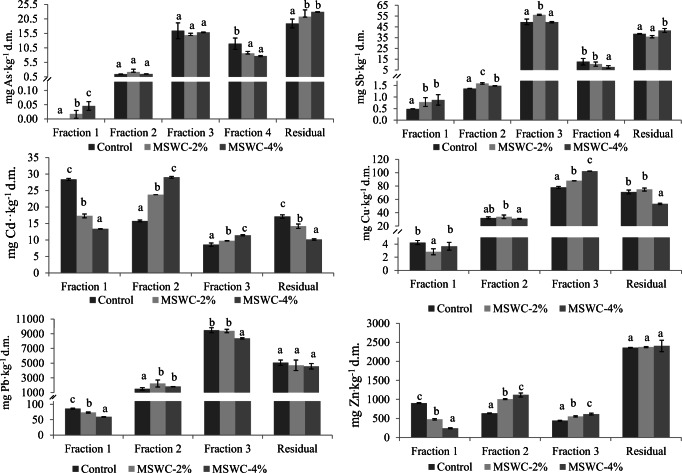
PTE released after sequential extraction. Values represent mean ± SD (*n* = 3). For each PTE and within each fraction, mean values followed by different letters indicate statistically significant differences according to the Tukey multiple comparison test (*P* < 0.05)

The total concentration of PTE in root-adhering soil did not significantly change among the treatments (Table 2). The results of the sequential extraction showed a relatively low content of water-soluble and exchangeable As and Sb in all the treatments (Fraction 1, Fig. 3), while the highest amount of such PTE extracted in Fraction 1 was found in MSWC-4% amended soils. These latter amounts only accounted for 1 and 0.1% of total As and Sb concentration respectively. The content of As exchanged by phosphate anions, i.e., that chemically bound to solid phase surfaces through inner-sphere complexes (Fraction 2, Fig. 3), was still low in all the samples and did not change significantly among the treatments. On the contrary, Sb detected in Fraction 2 slightly increased in the amended soils. Most of As and Sb were associated to poorly crystalline and amorphous Fe and Al (hydr)oxides (~ 30 and 48 % of the total As and Sb, Fraction 3). The addition of MSWC determined an increase of this fraction only for Sb in MSWC-2%. The As associated to well-crystallized Fe and Al(hydr) oxides (Fraction 4) decreased significantly in the soils treated with MSWC with respect to control soil, while Sb extracted in this fraction decreased significantly only in MSWC-4%. Sb and As strongly retained by the soil colloidal components and or precipitated (i.e., the residual fraction) were affected by MSWC addition: the residual As increased by 1.12- and 1.21-fold in MSWC-2 and MSWC-4% respectively compared with control soil, while the residual Sb increased only in MSWC-4% (Fig. 3). All this is expected to have a positive impact on soil microbiota and its functioning.

The water-soluble and readily exchangeable fraction of Cd, Cu, Pb, and Zn (Fraction 1), significantly decreased in amended soils, compared with control soil (with the exception of Cu in MSWC-4%; Fig. 3). It is remarkable the decrease of Cd detected in Fraction 1 of treated soils (− 39 and 53% in 2 and 4% amended soils respectively, compared with control soil). These results were interpreted in relation to the MSWC capacity to retain these PTE through specific adsorption mechanisms (Garau et al. 2019a), as well as to the pH increase in planted amended soils. The fraction of Cd, Cu, Pb, and Zn associated with carbonates and involved in weak surface complexes (Fraction 2) generally increased in amended soils (Fig. 3) and this was interpreted as the consequence of the increase of metal bicarbonates or carbonates in such soils (Khan et al. 2009). The relatively immobile, and not readily bioavailable or leachable Cd, Cu and Zn fraction (Fraction 3) increased in amended soils with respect to control (Fig. 3), while the opposite was found for Pb in MSWC-4% soil. The different behavior of PTE extractable with EDTA after plant growth and the lack of a single trend was also recorded by Pinamonti et al. (1997).

The residual fraction of cationic PTE varied depending on the MSWC rate and PTE considered (Fig. 3). After *C. cardunculus* growth the residual Pb and Zn was not significantly affected by MSWC addition. By contrast, after plant growth, residual Cd and Cu decreased in the amended soils; this may be linked with the activity of roots and soil microorganisms, likely enhanced by MSWC, which favored an increase of PTE not vey mobile, i.e., PTE pools extracted with EDTA or NaOAc, and a consequent decrease of residual PTE.

Taken together, these results showed that the combined effect of MSWC and *C. cardunculus* may favor a weak increase of labile PTE (especially for As and Sb) which nonetheless did not significantly contribute to increase the absorption of PTE by cardoon plants grown on amended soils.

### Influence of MSWC and cardoon growth on soil enzymatic activities and community-level physiological profile

**Fig. 4:**
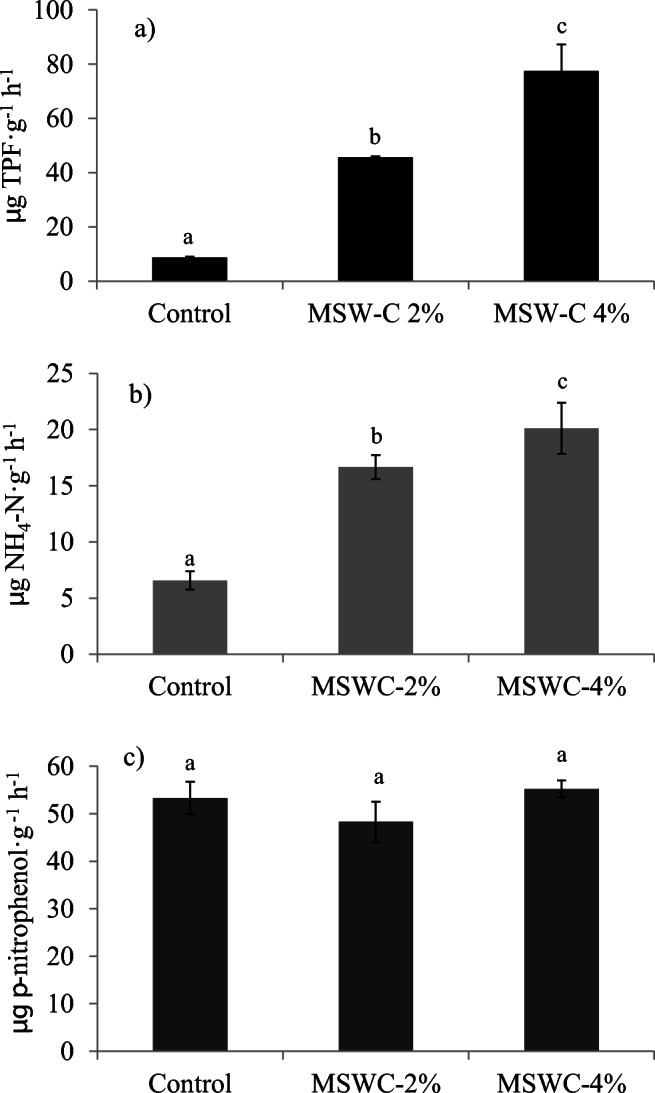
Dehydrogenase (DHG) (a), urease (URE) (b), and β-glucosidase (GLU) (c) activities in amended and unamended soils. Values represent mean ± SD (*n* = 3). For each enzyme activity, mean values followed by different letters indicate statistically significant differences according to the Tukey multiple comparison test (*P* < 0.05)

A suite of biochemical and microbial features were used as indicators of the biological impact of MSWC and *C. cardunculus* on PTE-contaminated soils. In particular, different soil enzymatic activities and Biolog community-level physiological profiles were determined after plant growth. Dehydrogenase (DHG), urease (URE), and β-glucosidase (GLU) activities are valuable indicators of the functional state of soils and are sensitive to environmental stress, hence PTE could influence these activities through enzyme denaturation or inactivation, by interfering with the enzyme-substrate complex and by exerting a direct toxic effects on soil microbiota (e.g., Abou Jaoude et al. 2019; Garau et al. 2019a, 2019b). After cardoon growth, DHG increased by 5.26- and 8.92-fold in MSWC-2 and MSWC-4% soils respectively, compared with control (Fig. 4a). This may be due to the combined effect MSWC addition and root activities, which could have enhanced the metabolism and the number of microorganisms, so increasing the DHG activities, in accordance with Garau et al. (2011, 2019a) and Garcia et al. (2002). In particular, compost DOC could have stimulated bacterial growth (and DGH activity) in amended soil as previously reported (Garau et al. 2017). Moreover, the better plant growth in the amended soils was reasonably accompanied by a greater exudation of C compounds from roots which eventually determined an increase of DHG (Walker et al. 2003).

URE activity, which catalyzes the release of CO_2_ and NH_3_ through the urea hydrolysis, was often used as an environmental stress indicator (Alvarenga et al. 2008). The URE activity was higher in amended soils, with respect to control (Fig. 4b). The MSWC addition, increasing the organic nitrogen content and the microbial population, could have favored a global increase of URE activity (Garau et al. 2017; Guo et al. 2019). As for DHG, the greater root exudation of C compounds in amended soils could have boosted soil microbial abundance and URE activity in such soils.

GLU, which is an extracellular enzyme involved in the carbon cycle (Alvarenga et al. 2008), was not affected by MSWC (Fig. 4c). GLU is mostly related to the abundance and activity of soil fungi (Zang et al. 2018), and apparently the C sources provided by the MSWC, as well as by root exudation, did not stimulate this microbial group. These results suggest that, as opposed to DHG and URE, β-glucosidase is not a reliable indicator of the functional recovery of polluted soils as previously observed (Garau et al. 2011, 2014).

**Fig. 5:**
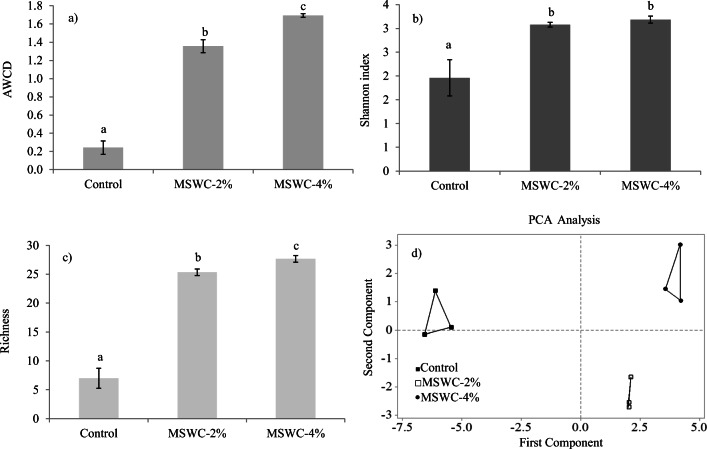
Average well color development (AWCD) (a), Shannon index (b), richness (c), and PCA plot of C source utilization data (d) of microbial communities extracted from amended and unamended soils. AWCD, Shannon index, and richness values represent mean ± SD (*n* = 3). For each Biolog-derived parameter, mean values followed by different letters denote statistically significant differences according to the Tukey multiple comparison test (*P* < 0.05)

Biolog community-level physiological profile (CLPP) was used to evaluate the influence of MSWC and cardoon on readily culturable soil bacteria. The average carbon source utilization (i.e., AWCD) of the different soil bacterial communities showed a significant increase in soils treated with MSWC (+ 6- and 7-fold in MSWC-2 and MSWC-4% respectively compared with control soil) (Fig. 5a). This indicated a higher potential metabolic activity of the bacterial populations in amended soils. Moreover, since AWCD is very often positively correlated with the size of heterotrophic bacterial populations (Garau et al. 2011, 2014, 2019b; Garland 1997), these results also suggested that the combination of MSWC and *C. cardunculus* can have a strong positive influence on the soil bacterial abundance. Importantly, this seems supported by the significantly higher DHG and URE activity observed after plant growth in amended soils (Fig. 4).

The Shannon index (H′), which provides information on the biological diversity of the microbial community (Farris et al. 2010; Pankhurst et al. 1997), increased by 1.57- and 1.63-fold in MSWC-2 and MSWC-4% respectively compared with control soil (Fig. 5b). The number of substrates oxidized by the microbial community (richness, S) followed the same trend; i.e., it increased by 3.62- and 3.95-fold in 2 and 4% amended soils respectively (Fig. 5c).

Taken together, AWCD, H′, and richness values indicated a clear impact of MSWC and cardoon on the catabolic potential and versatility of the soil bacterial communities inhabiting the polluted soil. As earlier mentioned, this could be due to the size increase of the bacterial community but also to a change of the community structure. To explore this latter possibility, C source utilization data were processed with PCA analysis (Fig. 5d). PCA, which accounted for approx. 82% of total variance in the first two components, clearly separated all the treatments, indicating an influence of both MSWC rate and *C. cardunculus* on the structure of soil microbial communities. Treatments were mainly separated by the first principal component (68.9 % of the total variance) which was highly correlated with the usage of selected C sources such as β-methyl-D-glucoside (*r* = 0.97), i-erythritol (*r* = 0.96), D-glucosaminic acid (*r* = 0.96), glucose-1-phosphate (*r* = 0.98), D-galactonic acid γ-lactone (*r* = 0.98), 2-hydroxy benzoic acid (*r* = − 0.70). Substrates mainly correlated with the second principal component (12.5 % of the total variance) were the following: α-cyclodextrin (*r* = 0.69), glycogen (*r* = 0.63), L-phenylalanine (*r* = 0.66), phenylethylamine (*r* = 0.63).

These differences, as well as those related to Biolog indexes (AWCD, H′, S), may be triggered by a reduced PTE environmental pressure in planted amended soils as previously pointed out by other studies (e.g., Garau et al. 2019a).

## Conclusions

The plant growth of *C. cardunculus* significantly increased after MSWC addition to a multi PTE-contaminated soil. Overall, this was explained with a reduced PTE mobility in amended soils, as well as with a general improvement of their physical-chemical properties.

The combined use of MSWC and cardoon plants produced a significant reduction of PTE bioaccumulation factors. However, the enhanced plant growth in amended soils determined significantly higher PTE mineralomasses, i.e., the total amount of PTE retained in plant tissues. This was particularly relevant for plant roots which appeared the main sink of PTE (except for Cd which accumulated in plant shoots).

After cardoon cultivation, a limited increase of labile As and Sb was recorded in amended soils which apparently did not negatively affect plant growth, while a positive influence of MSWC on C source usage and versatility was observed.

Overall, the results obtained indicate that the combined use of cardoon plants and MSWC could be useful for the aided phytoremediation of multi PTE-contaminated soils. However, further studies are needed to evaluate the stability over time of the observed effects as well as to assess the suitability of such phytoremediation strategy in field conditions.

## Electronic supplementary material

ESM 1 (DOCX 31 kb)
