# Supplementary material for: Evaluation of Cynara cardunculus L. and municipal solid waste compost for aided phytoremediation of multi potentially toxic element–contaminated soils
Source: Environ Sci Pollut Res Int. 2020 Sep 10;28(3):3253–65. doi: 10.1007/s11356-020-10687-2 (PMC7788029; doi:10.1007/s11356-020-10687-2)
Supplement: Supplementary file 1 — (DOCX 31 kb) [file 11356_2020_10687_MOESM1_ESM.docx]

**Table S1** Characteristics of the MSWC, untreated polluted (control) and MSWC-amended soils before the cardoon plants growth (dry matter basis). Values represent mean ± SD (n = 3). For each row mean values followed by different letters indicate statistically significant differences according to the Tukey multiple comparison test (*P* < 0.05)

|  | MSW-C | Control soil | MSWC-2% | MSWC-4% |
| --- | --- | --- | --- | --- |
| pH | 7.93 | 5.93^a^ | 6.53^b^ | 6.78^c^ |
| Electric Conductivity (EC, mS cm^−1^) | 3.26 | 1.34^a^ | 1.75^b^ | 2.07^c^ |
| Ash (%) | 42.05 | 91.63^a^ | 90.87^a^ | 90.10^a^ |
| Total organic matter (%) | 47.45 | 3.24^a^ | 3.99^b^ | 4.85^c^ |
| Total N (%) | 2.18 | 0.13^a^ | 0.17^b^ | 0.23^c^ |
| Total H (%) | 3.17 | 2.52^a^ | 2.55^ab^ | 2.70^b^ |
| Total C (%) | 27.46 | 1.88^a^ | 2.32^b^ | 2.81^c^ |
| Dissolved organic carbon (DOC, mg·g^−1^) | 0.817 | 0.11^a^ | 0.25^b^ | 0.48^c^ |
| P available (mg·kg^−1^ d.w.) | 62.24 | 22.62^a^ | 32.83^b^ | 44.82^c^ |
| Cation Exchange capacity (CEC, cmol_(+)_·kg^−1^) | 92.30 | 36.86^a^ | 39.01^ab^ | 42.04^b^ |
| pH_PCZ_ | 3.24 | 3.52^a^ | 5.24^b^ | 5.57^b^ |
| Exchangeable Na (cmol_(+)_·kg^−1^) | - | 0.44^a^ | 0.79^b^ | 1.24^c^ |
| Exchangeable K (cmol_(+)_·kg^−1^) | - | 1.03^a^ | 1.55^b^ | 2.09^c^ |
| Exchangeable Ca (cmol_(+)_·kg^−1^) | - | 22.84^a^ | 25.91^ab^ | 27.92^b^ |
| Exchangeable Mg (cmol_(+)_·kg^−1^) | - | 10.88^a^ | 9.72^a^ | 9.95^a^ |
| *Total trace elements (mg·kg^−1^)* |  |  |  |  |
| As | n.d. | 48.75^a^ | 47.29^a^ | 46.31^a^ |
| Cd | n.d. | 67.45^a^ | 65.43^b^ | 64.08^b^ |
| Cu | 19.24 | 181.45^a^ | 180.34^a^ | 179.44^a^ |
| Pb | 3.72 | 15,383^a^ | 15,690^a^ | 15,116^a^ |
| Sb | n.d. | 109.48^b^ | 106.20^a^ | 104.01^a^ |
| Zn | 30.52 | 4,076^a^ | 4,261^a^ | 4,082^a^ |

n.d. = not determined.
